# Analysis of menstrual effluent: diagnostic potential for endometriosis

**DOI:** 10.1186/s10020-018-0009-6

**Published:** 2018-03-19

**Authors:** Laura A. Warren, Andrew Shih, Susana Marquez Renteira, Tamer Seckin, Brandon Blau, Kim Simpfendorfer, Annette Lee, Christine N. Metz, Peter K. Gregersen

**Affiliations:** 1 The Donald and Barbara Zucker School of Medicine at Hofstra/Northwell, 500 Hofstra Blvd, Hempstead, NY 11549 USA; 2 Robert S. Boas Center for Genomics and Human Genetics, The Feinstein Institute for Medical Research, 350 Community Drive, Manhasset, New York, 11030 USA; 3 Seckin Endometriosis Center, 872 Fifth Avenue, New York, NY 10065 USA

**Keywords:** Decidualization, Biomarkers, Menstruation, Stromal fibroblast cells

## Abstract

**Background:**

Endometriosis is a chronic and underdiagnosed disease which affects 5–10% of women of childbearing age and is characterized by growth of endometrial tissue outside of the uterus, most often in the peritoneal cavity. Delay in diagnosis is a major problem for management of this disorder, and treatment is often not initiated until the disease has progressed for many years. Although the exact etiology of endometriosis remains unknown, retrograde menstruation is recognized as a common underlying factor leading to the deposit of menstrual effluent (ME) into the peritoneal cavity. Differences in the cellular biology and genetics of the cells within ME are therefore likely to explain why endometriosis develops in only a subset of women.

**Methods:**

Patients with and without endometriosis were consented to provide ME. ME was analyzed by flow cytometry for CD45- and CD45+ cell populations or used to isolate stromal fibroblast cells. ME-derived stromal fibroblast cells were assessed using decidualization assays following the addition of cAMP and IGFBP-1 concentrations in the culture supernatants were measured by ELISA. In addition, RNA was collected and analyzed by RNA-Seq and qPCR for markers of decidualization and to identify differentially expressed genes in ME-derived stromal fibroblast cells obtained from controls and subjects with endometriosis (±cAMP).

**Results:**

Flow cytometry analysis of cell subsets within the CD45+ fraction of ME revealed a significant decrease in the number of uterine NK cells in endometriosis patients compared with controls (*p* < 0.01). No other significant differences within either the CD45+ or CD45- cell populations were observed. Most strikingly, ME-derived stromal fibroblast cells cultured from endometriosis subjects showed impaired decidualization potential compared with controls. Highly significant differences in decidualization response were detected by measuring IGFBP-1 production at multiple time points after cAMP stimulation (*p* = 0.0025 at 6 h; *p* = 0.0045 at 24 h; *p* = 0.0125 at 48 h). RNA-Seq and qPCR analyses were used to identify genes differentially expressed by ME-derived stromal fibroblast cells obtained from endometriosis and control subjects.

**Conclusions:**

Menstrual effluent can be useful for investigating the pathobiology of endometriosis and for developing a non-invasive diagnostic for endometriosis which may lead to earlier and more effective treatments for this common disorder.

**Electronic supplementary material:**

The online version of this article (10.1186/s10020-018-0009-6) contains supplementary material, which is available to authorized users.

## Background

Endometriosis is a chronic disease characterized by the growth of endometrial-like tissue outside of the uterus. It affects 5 to 10% of women globally (Giudice, 2010a). This condition is defined clinically by its potential to cause pelvic pain, dysmenorrhea, and infertility (Giudice, 2010a). Annually endometriosis costs in the United States have been estimated to be as high as $50 billion due to medical expenses, loss of worker productivity, and both social and personal burdens (Simoens et al., 2012). One of the most challenging problems of endometriosis is the prolonged time between disease onset and definitive diagnosis by laparoscopic surgery, which is approximately 7 to 10 years (Hadfield et al., 1996). This delay in diagnosis and early treatment is caused, in part, by the lack of non-invasive diagnostic tests for endometriosis (Ballard et al., 2006; Giudice, 2010b).

Although the causes of endometriosis are not completely understood, one commonly accepted theory suggests that “retrograde menstruation” (the flow of menstrual effluent (ME) through the fallopian tubes into the peritoneal cavity) is a major contributing factor. Retrograde menstruation occurs in almost all women (Halme et al., 1984) and therefore other factors must contribute to the development of endometriosis. In this context, a key characteristic of ME is the presence of mesenchymal stem cells (MSCs) and stromal fibroblast cells (SFCs), which are phenotypically similar (Denu et al., 2016; Haniffa et al., 2009; Hematti, 2012; Ulrich et al., 2012), and are found within endometriosis lesions (Figueira et al., 2011) and eutopic endometrium (Barragan et al., 2016; Gargett, 2006; Masuda et al., 2010; Gargett, 2007; Fazleabas et al., 2002). MSCs and SFCs can be readily grown in culture and exhibit immunomodulatory and angiogenic properties, making them a potential therapeutic or regenerative resource for a variety of conditions, such as cardiac ischemia, limb ischemia, stroke, and lung injury (Zhang et al., 2013; Meng et al., 2007; Vu et al., 2015; Borlongan et al., 2010; Rodrigues et al., 2016).

Previous studies report that SFCs isolated and cultured from endometrial biopsies have the capacity to decidualize (Barragan et al., 2016). Decidualization is a normal process within the mid-secretory phase during which endometrial stromal cells differentiate into specialized secretory decidual cells required for successful embryo implantation and placental development (Gellersen & Brosens, 2014; Dunn et al., 2003; Ramathal et al., 2010). Giudice and colleagues have clearly shown that SFCs derived from endometrial biopsies obtained from patients with endometriosis display impaired decidualization ex vivo when compared to endometrial biopsy-derived SFCs obtained from control subjects (Barragan et al., 2016). Thus, we postulated that ME-derived SFCs may offer a non-invasive resource for the early diagnosis of endometriosis based on their phenotypic differences and decidualization capacity. In addition, we explored whether other phenotypic characteristics of ME-derived cells could be informative for diagnosing and better understanding the pathobiology of endometriosis, as well as other reproductive disorders. To enable these goals we established a protocol for the collection and transport of ME for investigative studies.

## Methods

### Subject recruitment and enrollment

Endometriosis subjects were recruited through the Research OutSmarts Endometriosis (ROSE) study (https://www.feinsteininstitute.org/rose-research-outsmarts-endometriosis/; IRB#13-376A) and control subjects were recruited from the Genotype and Phenotype (GaP) registry (http://www.feinsteininstitute.org/robert-s-boas-center-for-genomics-and-human-genetics/gap-registry/; IRB#13-627A); both studies were approved by the Institutional Review Board (IRB) of Northwell Health. Briefly, women of reproductive age who were not pregnant or breast-feeding, did not use an internal birth control device (e.g. IUD) and were menstruating and willing to provide ME sample(s) were recruited and consented. Women who reported the diagnosis of endometriosis, as determined by laparoscopic surgery and were at least one year post-op following endometrial lesion removal, were recruited via the ROSE study (22 to 43 years old). Control subjects who self-reported no history suggestive of a diagnosis of endometriosis were recruited through the GaP registry (21 to 47 years old). For functional decidualization experiments using ME from endometriosis patients and controls, only subjects who reported that they were not using hormonal birth control were included.

### Collection of menstrual effluent (ME)

ME was collected for approximately 6–10 h per collection on days 0, 1, or 2 of the menstrual phase, where day 0 is the first day of the menstrual cycle, using the DivaCup (a menstrual cup produced by Diva International Inc., Ontario, Canada) as described previously (Howard et al., 2011). ME was transferred to a sterile 50 mL conical tube containing 1 mg Normocin™ (Invivogen, San Diego, CA) and 500 units penicillin streptomycin (Gibco), placed in a refrigerated box and transported by express shipment to the lab within approximately 24 h. In pilot experiments we observed no differences in freshly obtained ME vs. 24 h refrigerated/shipped ME, based on cellular composition determined by flow cytometry, viability of non-CD66+ cells by flow cytometry, or isolation/culture success of ME-derived SFCs. These observations are consistent with those of van der Molen and colleagues, who reported that CD45+/CD66- ME cells remained viable for at least 72 h post collection (van der Molen et al., 2014).

### Flow cytometry of ME and cultured SFCs

After removing the soluble fraction from whole ME following a brief centrifugation and lysis of red blood cells with BD Pharm Lyse (BD Biosciences, Billerica, MA), samples (from *n* = 8 endometriosis and *n* = 14 control subjects) were passed through a 35 μm cell strainer attached to polystyrene FACS tube (Thermo Fisher Scientific, Bridgewater, NJ). ME-cells were then pelleted and incubated with Human Fc Block (BD Biosciences) for 20 min. After a brief wash, cells were incubated with either the CD45+ or CD45- panels of antibodies listed in Table 1 or the appropriate isotype control antibodies. All cells were subjected to Aqua live/dead viability staining (Thermo Fisher) and then stained with antibodies in Table 1 for 30 min at 4 °C. The cells were washed twice with 1% FBS in PBS and fixed with 3% paraformaldehyde (PFA) if not being analyzed same day. All data were collected on the Fortessa  Flow Cytometer (BD) and analyzed using FlowJo software (version 10.1r5; Ashland, OR).

**Table 1:** ME FACS Staining Panels

Fluorochrome	Antigen	Cat. No
CD45+ Staining Panel		
PE	CD20	555623
FITC	CD16	302006
APC	CD56	555518
Pacific Blue	CD66b	305112
APC-Cy7	CD3	557757
PerCP-Cy5.5	CD14	562692
PE-Cy7	CD45	557748
CD45- Staining Panel		
PE	CD105	560839
PerCP-Cy5.5	CD326	347199
APC	CD73	560847
FITC	CD90	555595
APC-Cy7	CD31	563653
PE-Cy7	CD45	557748
Aqua (AmCyan)	Live/Dead	L34966
CD45+ Staining Panel Isotype Controls		
PE	Mouse IgG_2b_, κ	555743
FITC	Mouse IgG_1_	349041
APC	Mouse IgG_1_, κ	555751
Pacific Blue	Mouse IgM, K	401619
APC-Cy7	Mouse IgG1, K	557873
PerCP-Cy5.5	Mouse IgG2b, K	558304
PE-Cy7	Mouse IgG_1_	348798
CD45- Staining Panel Isotype controls		
PE	Mouse IgG_1_	349043
APC	Mouse IgG_1_, κ	555751
PerCP-Cy5.5	Mouse IgG_1_, κ	552834
FITC	Mouse IgG_1_	349041
APC-Cy7	Mouse IgG1, K	557873
PE-Cy7	Mouse IgG_1_	348798

Cultured SFCs (passage 1 from *n* = 7 endometriosis and *n* = 7 control subjects), as described below, were lifted with ACCUTASE™ (STEMCELL TECHNOLOGIES, Cambridge, MA) at confluence and resuspended in Brilliant Stain Buffer (BD Biosciences) with the staining antibody panels listed in Table 2 or appropriate isotype control antibodies for 30 min at 4 °C and fixed with 2% PFA. The cells were washed twice with 1%FBS in PBS. All data were collected on the Fortessa Flow Cytometer (BD) and analyzed using FlowJo software (version 10.1r5; Ashland, OR).

**Table 2:** ME-SFC Staining Panel

Fluorochrome	Antigen	Company	Cat. No.
PERCP-Cy5.5	CD105	BD Pharmingen	560819
FITC	CD90	BD Pharmingen	555595
APC	CD73	BD Pharmingen	560847
BV421	CD140b	BD Pharmingen	564124
PE	SUSD2	Biolegend	327406
BV711	CD146	BD Pharmingen	563186
BV786	CD45	BD Pharmingen	563716
BV605	EPCAM	Biolegend	324224
PERCP-Cy5.5	Isotype	BD Pharmingen	552834
FITC	Isotype	BD Pharmingen	349041
APC	Isotype	BD Pharmingen	555751
BV421	Isotype	BD Pharmingen	562439
PE	Isotype	Biolegend	400114
BV711	Isotype	BD Pharmingen	563044
BV786	Isotype	BD Pharmingen	564230
BV605	Isotype	Biolegend	400350

### ME-derived stromal fibroblast cell (SFC) isolation and culture

Whole ME (500 μL) was plated in T75 flasks in SFC media: 10% FBS (MSC-qualified, Gibco), 1X glutamine (Gibco), 100 units/ml penicillin-streptomycin (Gibco), and 100 μg/ml Normocin™ in DMEM (Gibco). Flasks were incubated at 37 °C/5%CO_2_ for 48–72 h in order to isolate SFCs by adherence. After washing, adherent cells were cultured to 80% confluence before lifting with trypsin/EDTA and subsequently freezing stock cells at passage 1 (p1).

### Decidualization assays using ME-derived SFCs

Cryopreserved ME-derived SFCs (p1) were defrosted and plated in 24-well plates in SFC media; at confluence SFCs (p2) were incubated at 37 °C/5%CO_2_ in decidualization media (2% FBS MSC-qualified, 1X glutamine, 100 units/ml penicillin-streptomycin, and 100μg/ml Normocin™ in Phenol Red Free DMEM with either 0.5 mM 8-Bromoadenosine 3′,5′-cyclic monophosphate (cAMP, Sigma-Aldrich, St. Louis, MO) ± 10 nM 17-beta estradiol (E2) (Tocris, Minneapolis, MN) and 1 μM medroxyprogesterone acetate (MPA, Sigma-Aldrich) or vehicle (PBS for studies with cAMP alone or 0.01% ethanol for studies with cAMP + E2 + MPA). Cells were stimulated for 6 h, 24 h, and 48 h, at which point supernatants and cell lysates were collected from each well for the time course experiment. Supernatants were analyzed for IGFBP-1 concentrations by ELISA (Duoset, R&D Systems, Minneapolis, MN) and results were normalized to cell lysate protein concentrations using the Bio-Rad Protein Assay Kit (Bio-Rad, Hercules, CA).

### RNA-Seq and qPCR

Total cellular RNA was isolated following stimulation of ME-derived SFCs (collected from *n* = 7 endometriosis subjects and *n* = 7 control subjects) with either 0.5 mM cAMP or vehicle for 6 h, as described above using the mirVana™ miRNA Isolation Kit and treated with the DNA-free™ kit (Ambion) to remove DNA (Ambion). Supernatants were also collected and analyzed for IGFPB-1 by ELISA, as described above. RNA sequencing was performed using the Illumina mRNA TrueSeq Stranded method. The raw image files from the NextSeq sequencer were de-multiplexed and converted to FASTQ files using Illumina’s bcl2fastq BaseSpace App. The FASTQ files were aligned to the hg19 human reference genome from GENCODE using the STAR2 aligner and the digital gene counts quantified using HTSeq. The differential expression analysis was done using DESeq2.

*ALDH1A1* expression was assessed by qPCR using RNA collected from ME-SFCs following 6 h treatment with vehicle or 0.5 mM cAMP (*n* = 7 endometriosis subjects and *n* = 7 control subjects). qPCR was performed with probes and primers designed using ProbeFinder online software (left primer: ccaaagacattgataaagccataa; right primer: cacgccatagcaattcacc) and probe #82 of the Universal ProbeLibrary (Roche, Basel, Switzerland). qPCR, using *GAPDH* as control, was performed on the Roche LightCycler 480 qPCR machine using the following conditions: 2 min at 50 °C, 10 min at 95 °C, followed by 40 cycles of 15 s at 95 °C and 1 min at 60 °C. Relative changes in gene expression were calculated as fold-changes using the comparative Ct (∆∆Ct method) and statistical analysis was done through the Mann-Whitney Test (Cikos & Koppel, 2009).

### Statistical analyses

#### Flow cytometry analyses of the cellular composition of whole ME

All analyses were carried out separately for each cell population. For each cell population within the CD45+ and CD45- subsets, a linear mixed model was used to examine the association between group and that population using SAS Version 9.4 (SAS Institute Inc., Cary, NC). The mixed models approach was used to account for the hierarchical nature of the data (namely, multiple samples from one or more cycles within a subject). Time of day (evening, overnight) and day of cycle collection was started (0, 1–2) were included as covariates in the model to adjust for any effects they might have on cell population.

Transformations (namely log or arcsine square root) were used to better meet the assumptions of the linear model. However, as the results obtained from the models using transformed data did not differ qualitatively from those using the raw data, results using the raw data are presented for ease of interpretation*.*

#### SFC Decidualization time course

To determine differences in IGBFP-1 protein levels between treatments and cases/controls, a Bayesian hierarchical model was implemented. The data consists of normalized measurements of IGFBP-1 protein levels of 7 endometriosis cases and 7 controls, across four different treatments (cAMP, vehicle for cAMP alone, ethanol vehicle, and cAMP+MPA + E2). Each treatment has protein measurements for all the subjects at three different time points (6 h, 24 h, and 48 h).

The response variable - protein levels - was log-transformed in order to adjust for heteroscedasticity in the original scale and the covariate included was an indicator variable for whether a subject was a case or a control, and indexed according to the treatment and time period in which the subjects were analyzed. For the hierarchical structure, subjects were nested within treatments at each time point. A model assuming normally distributed residuals estimated both the mean protein content at each level; a group parameter was used to estimate the effect of being in the case/control group. The posterior probability of the group effect being zero was assessed and is summarized in Additional file 1: Table S1. Data was arranged using R (version 3.3.2) and the model was implemented in Stan (version 2.16.2) using the R interface. The model is estimated using the No-U-Turn sampler algorithm (Hoffman & Gelman, 2014) a version of Hamiltonian Monte Carlo (Neal, 1994). Once posterior draws were estimated, the model was simulated for each draw, resulting in the generated quantities. All other data were analyzed using the Mann-Whitney test.

## Results

### ME contains CD45+ and CD45- cell populations and ME collected from endometriosis subjects has fewer uterine NK cells

**Fig. 1:**
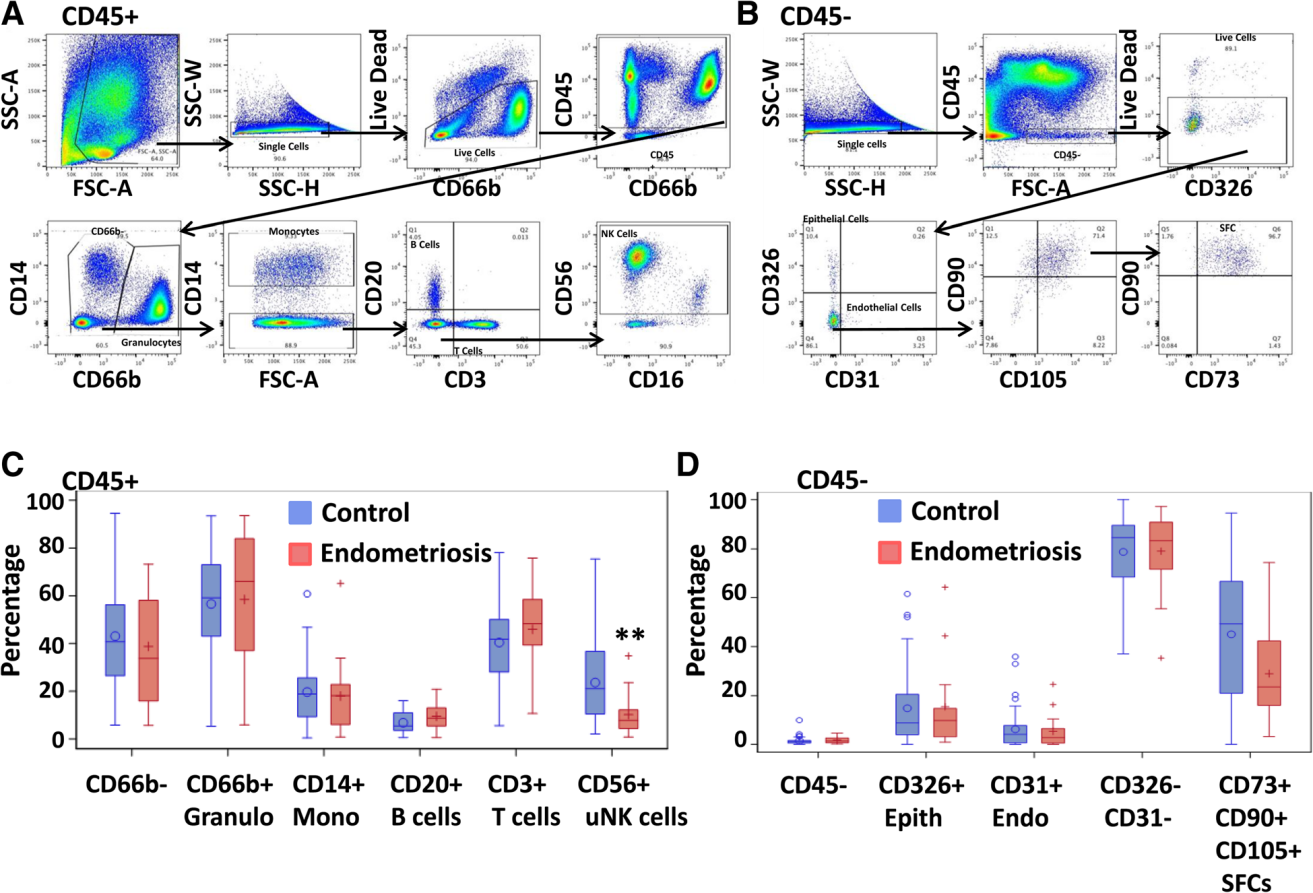
Flow Cytometric analysis of ME from endometriosis and control subjects CD45+ (*n* = 14 control, *n* = 8 endometriosis) (**a**) and CD45- (*n* = 14 control, *n* = 6 endometriosis) (**b**) flow cytometry staining scheme for ME. Box plots showing the cellular composition CD45+ subsets (CD66b + granulocytes [Granulo], CD14+ monocytes [Mono], CD20+ B cells, CD3+ T cells, and CD56+ uterine natural killer (uNK) cells) (**c**) and CD45- subsets (CD45-, CD326+ epithelial cells [Epith], CD31+ endothelial cells [Endo], CD326-/CD31- cells, and CD73+/CD90+/CD105+ [SFCs] (**d**) found in menstrual effluent from women with and without endometriosis. The CD66b + and CD66b- populations were normalized to the CD45+ population cell counts and the CD14+ Mono, CD20+ B Cells, CD3+ T cells, and CD56+ uNK cell populations were normalized to CD66b-population cell counts. The Epith, Endo, CD326-/CD31- cells, and SFC populations were normalized to the CD45- population cell counts. Data are shown as box plots depicting the median and interquartile ranges for each cell subset; significance for uNK cells **p = 0.01 (**c**)

Flow cytometric analysis was performed on fresh ME collected from control and endometriosis subjects for both CD45+ leukocyte populations and CD45- cell populations (Fig. 1a, b). After gating out dead cells, ME is mainly comprised of CD45+ cells, with the CD45- cell populations contributing on average 1.6 ± 0.3% for control subjects and 1.9 ± 0.5% for endometriosis subjects (Additional file 1: Table S1). The CD45+ population includes granulocytes, monocytes, T cells, B cells, and natural killer (NK) cells. The vast majority of NK cells found in ME are uterine NK (uNK) cells based on their lack of CD16 expression and high expression of CD56 (see Fig. 1a). Depending on the ME sample the CD66b + population made up the majority of the CD45+ population, with 55.6 ± 5.1% for control subjects and 56.7 ± 6.5% for endometriosis subjects (Additional file 1: Table S1). The CD66b + granulocyte population was variable between subjects and samples collected, possibly due to the poor viability of granulocytes. In order to account for the variability of the granulocyte population between samples, the data was normalized by adjusting the cell count of the population of interest (monocytes, T cells, B cells, or uNK cells) to the cell count of the CD45+ CD66b- population. Although, no significant differences between ME collected from endometriosis vs. control subjects were found for granulocytes, monocytes, T cells, or B cells, there were significantly less uNK cells (*p* = 0.01) in ME collected from endometriosis subjects compared to control subjects (Fig. 1c).

The CD45- populations analyzed included epithelial cells, endothelial cells, and stromal fibroblast cells (SFCs) (Fig. 1b). There were no significant differences found in the percentages of epithelial cells (CD31-, CD326+/EPCAM) or endothelial cells (CD31+, CD326-) among total CD45- cells when comparing ME obtained from control to endometriosis subjects (Fig. 1d). The SFC population was defined as: CD45-/CD326-/CD31-/CD90+/CD105+/CD73+ (Fig. 1b, d) and no significant differences in the SFC populations were found when comparing fresh ME obtained from endometriosis subjects to control subjects (Fig. 1d).

**Fig. 2:**
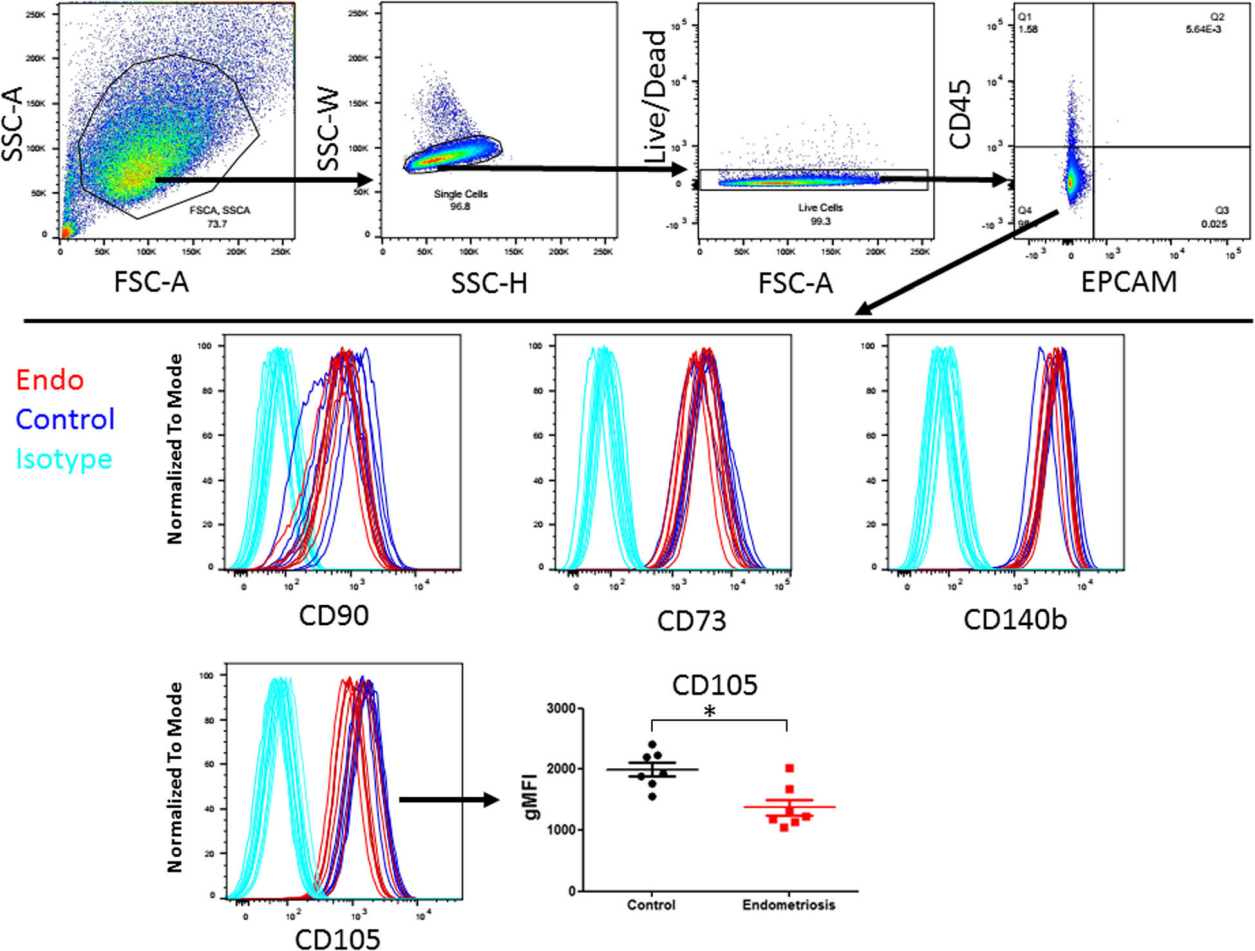
Cultured ME-derived SFCs express CD73, CD90, CD105, and CD140b but not CD45 or CD326. Flow cytometry gating of cultured ME-derived SFCs to show CD45-/CD326- population (upper panel). The CD45- population of ME derived SFCs from endometriosis (*n* = 7) and control (*n* = 7) subjects at passage 1 were further analyzed by flow cytometry for CD90, CD73, CD140b, and CD105 expression (lower panel). CD105 expression is lower on endometriosis-SFCs when compared to control subject-SFCs (lower right panel). Data are shown as geometric mean fluorescence intensity (gMFI), for each subject’s SFC culture and the horizontal lines are the group means, *p = 0.03

Next, we assessed cultured ME-derived SFCs (p1) for surface markers by flow cytometry. Cultured SFCs were positive for CD105, CD90, and CD73 and negative for CD45 (Fig. 2). In addition, ME-SFCs expressed the fibroblast marker CD140b (Fig. 2). No significant differences in CD90 or CD73 expression (based on geometric mean fluorescence intensity, gMFI) were found when comparing cultured ME-SFCs derived from endometriosis to control subjects. However, CD105 expression (gMFI) was significantly higher on control subject SFCs than endometriosis subject SFCs (*p* < 0.05) (Fig. 2 lower right panel).

### Cultured ME-derived SFCs from endometriosis subjects have reduced decidualization capacity

**Fig. 3:**
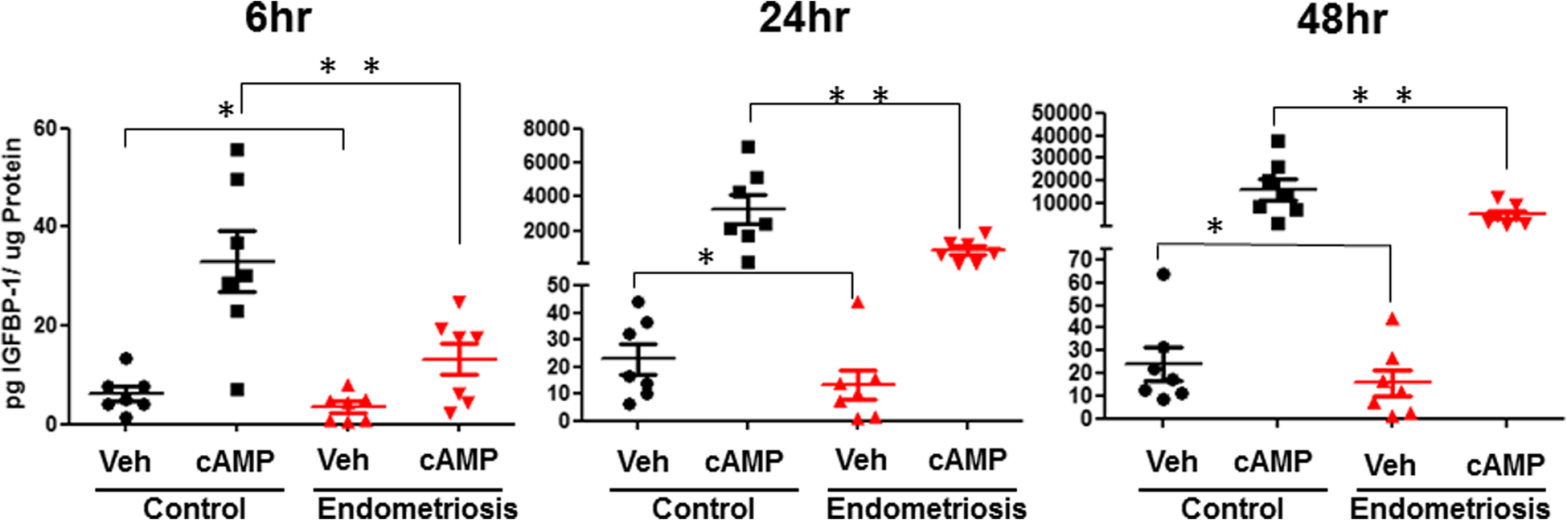
ME-derived SFCs obtained from endometriosis subjects exhibit reduced decidualization capacity. Time course of IGFBP-1 secretion by vehicle (Veh) and cAMP treated (0.5 mM) ME-derived SFCs isolated from endometriosis and control subjects (*n* = 7 control, *n* = 7 endometriosis). Data are shown as IGFBP-1 values for each subject’s SFC culture and the horizontal lines represent group means. ** posterior probabilities (Pr) < 0.01 *Pr < 0.05. Statistics were performed on log transformed data, as described in the methods, see Additional file 1: Table S3)

It has been reported that endometrial stromal cells cultured from endometrial biopsies and hysterectomy samples obtained from endometriosis subjects have a reduced potential to decidualize when compared to those obtained from control subjects (Barragan et al., 2016; Klemmt et al., 2006). In order to investigate whether similar differences are observed using cultured SFCs derived from ME, we performed in vitro decidualization assays at 3 different time points: 6 h, 24 h, and 48 h post 0.5 mM cAMP vs. vehicle using passage 2 SFCs obtained from control and endometriosis subjects. Decidualization was assessed by measuring IGFBP-1 concentrations in the culture supernatants by ELISA. As expected, stimulation with cAMP induced decidualization of ME-SFCs, as determined by enhanced IGFBP-1 production, which was detectable at 6 h and continued to increase through 48 h (Fig. 3). Significantly less IGFBP-1 was produced following both vehicle-treatment and cAMP-treatment at all three time points by ME-SFCs obtained from endometriosis subjects when compared to control subjects (*p* < 0.05) (Fig. 3). Similarly, the addition of MPA and E2 to cAMP induced decidualization, as determined by IGFBP-1 levels. ME-SFCs obtained from endometriosis subjects produced significantly less IGFBP-1 following stimulation when compared to control ME-SFCs (Additional file 2: Figure S1). Additionally, individual subject’s ME-SFCs that produce high or low amounts of IGFBP-1 do so consistently across all three-time points (Additional file 3: Figure S2).

### Gene expression analysis of cultured ME-SFCs derived from endometriosis subjects and controls

**Fig. 4:**
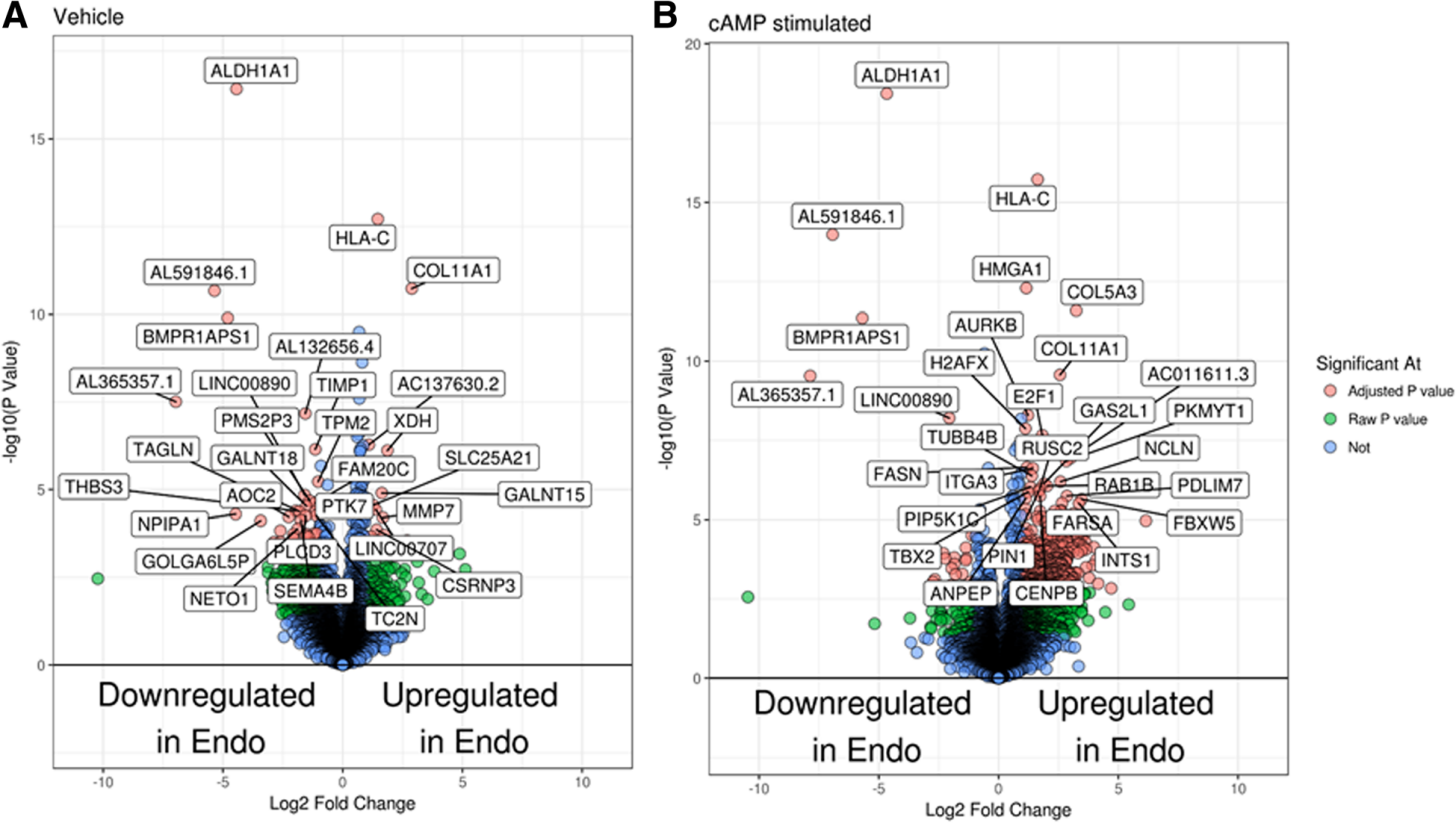
Numerous genes are differentially expressed when comparing vehicle-treated and cAMP-treated SFCs obtained from subjects with endometriosis vs. controls. Volcano plots of genes differentially regulated between endometriosis and control subjects in the vehicle-treated group (**a**) and the cAMP-treated group (**b**) (*n* = 3 control, *n* = 3 endometriosis for A and B)

**Fig. 5:**
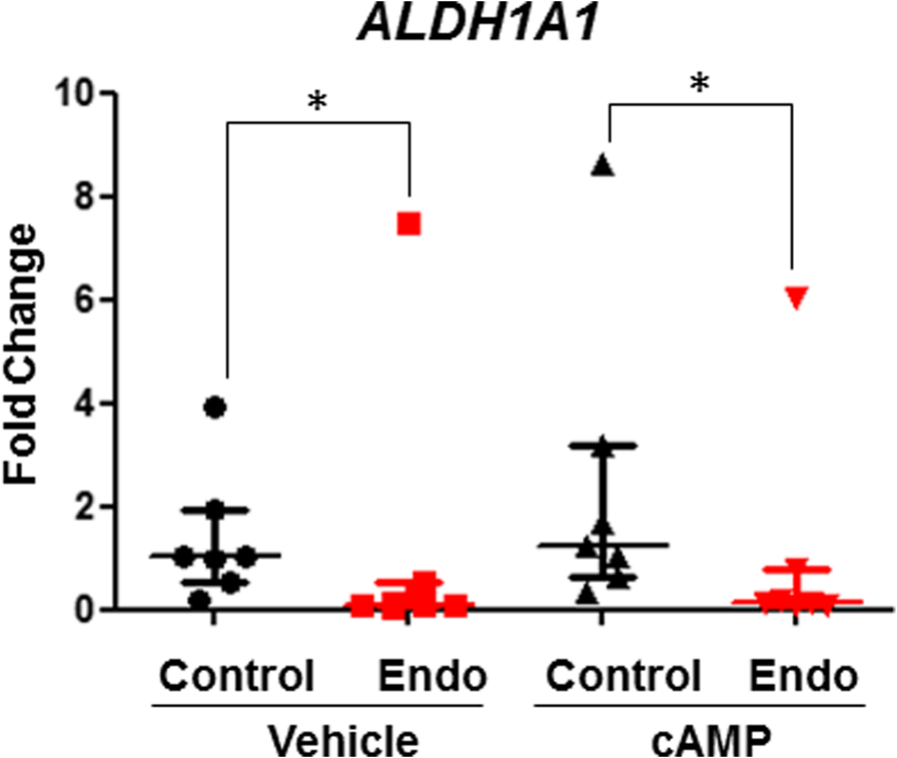
SFCs from endometriosis subjects exhibit reduced *ALDH1A1* expression. *ALDH1A1* expression by vehicle and cAMP-treated SFC obtained from subjects with and without endometriosis (*n* = 7 subjects per group) was confirmed by qPCR. Data are shown as relative gene expression (fold-change) for each subject’s SFC culture; horizontal lines represent group medians and vertical lines represent interquartile ranges, *p < 0.05, calculated using Mann Whitney Testᅟ

We performed RNA-Seq on cultured ME-SFCs isolated from a small number of endometriosis (*n* = 3) and control (*n* = 3) subjects following cAMP or vehicle-treatment for 6 h (Fig. 4a, b). Many of the genes previously reported to be increased during decidualization were also increased in the decidualizing SFCs from both endometriosis subjects and controls (Fig. 4b; Additional file 1: Table S2). These genes included *IGFBP1*, *SST*, *PRL*, *BCL2L11*, *WNT5A*, and *FOXO1* (Additional file 1: Table S2). When comparing ME-SFCs obtained from endometriosis and control subjects, we observed differentially regulated genes in both vehicle-treated and cAMP-treated cultures. A volcano plot shows the top differentially regulated genes for both endometriosis and control subjects in the cAMP-treated and vehicle-treated groups (Fig. 4a, b). The most striking difference between the control and endometriosis SFCs was the high expression of *ALDH1A1* (encoding aldehyde dehydrogenase 1 family member A1) in control SFCs, regardless of treatment. We confirmed these findings using qPCR for a larger number of ME-SFCs (*n* = 7 subjects per group), as shown in Fig. 5. These differences were unaffected by stimulation with cAMP (Fig. 5). Although the relative lack of expression in the endometriosis SFCs remained significant, there was an outlier in the endometriosis group which was not readily explained by any particular subject characteristic.

## Discussion

Almost all women experience retrograde menstruation, a process by which menstrual effluent (ME) shed from the endometrium is effluxed through the fallopian tubes into the peritoneal cavity, where it implants on the serosal surface (Halme et al., 1984). Evidence that effluxed ME is likely to be a major source of cells responsible for the formation of peritoneal endometriosis lesions in patients is supported by studies in baboons showing that consecutive intraperitoneal injections of shed menses results in endometriosis lesions in the peritoneal cavity and by the presence of spontaneous endometriosis in menstruating non-human primates, along with the complete absence of endometriosis in non-menstruating mammals (Fazleabas et al., 2002; D'Hooghe et al., 1994; D'Hooghe et al., 2009). Our findings suggest that analysis of ME shed from the endometrium can be harnessed to investigate the pathobiology of endometriosis and to develop early, non-invasive diagnostic methods for endometriosis.

Individual differences in the cellular content of ME offer a potential tool for investigating why only 5–10% of females actually develop endometriosis, given the ubiquity of retrograde menstruation. A relatively small number of published studies have analyzed the composition of ME, comparing it to that of peripheral blood; these studies primarily focused on phenotyping the CD45^+^ cell populations (van der Molen et al., 2014; Hosseini et al., 2015). Consistent with previous reports we found that ME is mainly comprised of CD66b + granulocytes, followed by CD3+ lymphocytes, CD56^bright^ uNK cells, and then CD14+ monocytes and CD20+ B cells (van der Molen et al., 2014; Hosseini et al., 2015). The variability that we observed among the various subpopulations of CD45+ cells (Fig. 1a  and c) was similar to that previously reported (van der Molen et al., 2014; Hosseini et al., 2015). Neither of these previous studies compared ME obtained from endometriosis subjects to controls. Despite the variability in the cellular composition of ME, we found that endometriosis subjects had significantly lower percentages of uNK cells when compared to controls (Fig. 1c). uNK cells are CD56^bright^/CD16-, unlike peripheral blood NK cells which are CD56^dim^/CD16+. uNK cells are proposed to play an important role in endometrial vascularization and decidualization, as well as the development of decidualization-related infertility observed in women with endometriosis (Gellersen & Brosens, 2014).

Decidualization is a process in which endometrial stromal fibroblast-like cells differentiate into specialized secretory decidual cells during the mid-secretory phase to prepare the uterus for implantation and placental development (Gellersen & Brosens, 2014; Dunn et al., 2003; Ramathal et al., 2010). A very recent study reveals that uNK cells target and eliminate senescent endometrial stromal fibroblast-like cells, and this may be an important process for successful decidualization (Brighton et al., 2017). A reduction in uNK cells found in ME collected from subjects with endometriosis may be linked to the impaired elimination of senescent uterine cells that compromises the decidualization process – which is implicated in the infertility observed in patients with endometriosis. Future studies will focus on more detailed analyses of the NK cell population found in ME collected from controls and subjects with endometriosis.

Several previous studies have highlighted the regenerative capacity of ME-derived CD45- mesenchymal and stromal cells (Zhang et al., 2013; Meng et al., 2007; Vu et al., 2015; Borlongan et al., 2010; Rodrigues et al., 2016). The CD45- cell fraction contributes to approximately 1.5–2% of the total non-RBC portion of ME. The majority of these cells were not endothelial or epithelial cells, but rather CD73/CD90/CD105 expressing cells. We observed no significant differences in these subpopulations of CD45- cells found in ME collected from endometriosis subjects vs. controls (Fig. 1d). However, we did not include a distinction between the endometrial MSC and SFC populations in the ME; this could be addressed in future studies using CD146/CD140b markers, as described by studies of endometrial biopsies (Barragan et al., 2016). Among cultured SFCs, we did observe a lower level of expression of CD105 (endoglin) in those isolated from ME collection from endometriosis patients. CD105 is an auxiliary receptor for the TGFβ receptor complex and regulates the binding of various ligands, including TGFβ1 and TGFβ3, as well as BMP-2 and -7 (Guerrero-Esteo et al., 2002). Differences in CD105 expression between endometriosis patients and controls have been reported by others (Hayrabedyan et al., 2005), but these studies focused on CD105 expression by the microvasculature of eutopic endometrium and ovarian endometriosis and not non-vascular CD105 expression. This finding will need to be explored further in future analyses.

A major innovation of this study over prior studies profiling the cellular composition of ME is that we included functional assessment of the SFCs (of the CD45- fraction). Taking advantage of the fact that defects in stromal cell decidualization (obtained from invasive uterine biopsies) have been reported in the setting of endometriosis (Klemmt et al., 2006; Aghajanova et al., 2011; Yin et al., 2012), we compared ME-derived SFCs obtained from controls and endometriosis subjects for their decidualization response using cAMP. We demonstrate that a reduced decidualization response by ME-derived SFCs is clearly associated with endometriosis, as shown in Fig. 3. In fact, ME-SFCs obtained from women with endometriosis exhibit significantly reduced IGFBP-1 expression in the absence of decidualization stimuli (Fig. 3). These differences persist over time in culture and likely reflect a quantitative impairment in decidualization capacity. This difference is observed with cAMP alone, as well as when decidualization is induced in the presence of cAMP + MPA + E2 (Additional file 2: Figure S1).

Interestingly, we have occasionally observed impaired decidualization capacity in control subjects’ SFCs (Fig. 3). This may reflect the presence of asymptomatic endometriosis in these individuals. Alternatively, genetic variation may influence the decidualization response in the absence of endometriosis. Genetic differences play a significant role in risk for endometriosis; a recent meta-analysis of over 170,000 endometriosis patients and almost 200,000 controls revealed 14 genetic regions of interest, including *WNT4*, *GREB1*, *IL1A*, and *CDKN2B* (Sapkota et al., 2017). The examination of phenotypic diversity of ME may also shed light on the mechanisms of genetic predisposition (Rahmioglu et al., 2015). In this context it will be of interest to examine decidualization capacity in control subjects who carry genetic risk alleles for endometriosis (Rahmioglu et al., 2015). For example, *WNT4* plays a direct role in the decidualization response, and *WNT4* risk haplotypes for endometriosis have been reported to contain an estrogen response element that may regulate its expression (Zhang et al., 2017). Over the last decade, we have established a large registry of normal controls (GaP) with GWAS genotype data and subjects can be recalled for phenotypic studies based on a genotype of interest (Gregersen, 2009; Gregersen et al., 2015). This offers the possibility of examining a variety of endometriosis risk haplotypes with regard to their impact on decidualization capacity in various populations. The use of ME as a source of SFCs makes such studies possible without the need for invasive procedures. Thus, we believe that one of the major benefits of this study is to open up the possibility of carrying out population-based genotype-phenotype studies that are relevant to better understanding the underlying pathogenesis of endometriosis.

Another potential benefit of studying ME, which can be sampled throughout menstruation and monthly, is that it may lead to the development of early, non-invasive diagnostic methods. The long delay between symptom onset and diagnosis in this disease is well established (Hadfield et al., 1996; Arruda et al., 2003), and no laboratory diagnostic makers have emerged to replace, or even guide, the performance of the gold standard of laparoscopic surgery and histologic confirmation. The expression of *BCL6* (Evans-Hoeker et al., 2016) has been reported to be altered in secretory phase endometrial biopsies and may be useful diagnostically, but this approach will require invasive biopsy procedures. Other possible diagnostic markers for endometriosis include CA-125 (Hirsch et al., 2017), VEGF, microRNAs, immunologic markers, and soluble ICAM levels (Acimovic et al., 2016; Vodolazkaia et al., 2016; Gagné et al., 2003; Matalliotakis et al., 2001; Kuessel et al., 2017; Mosbah et al., 2016; Bohler et al., 2007; Drosdzol-Cop et al., 2012; Wu et al., 2015); however, none of these can identify endometriosis patients with both high sensitivity and specificity. It is clear that the need for a non-invasive diagnostic marker or method remains, and it would appear that the analysis of ME may offer a path to develop such tests. As discussed above, our findings of a reduced uNK cell population in the ME of endometriosis subjects may not be specific for endometriosis, and may simply reflect the relative lack of decidualization in these subjects, as decidualization is accompanied by extensive uNK infiltration during the mid-luteal phase under normal conditions (Gellersen & Brosens, 2014). Future studies are warranted to investigate the mechanism underlying this difference.

Although we observed significant decidualization by ME-derived SFCs (based on IGFBP-1 protein release) within 6 h of cAMP simulation, a diagnostic based on culturing ME-derived stromal cell populations to assess decidualization capacity is not practical for routine clinical use. We believe that a direct assessment of freshly isolated stromal cells from ME is likely to be the most promising approach, based on gene expression, epigenetic analysis or other phenotypic changes in these cells. The fact that gene expression differences, such as *ALDH1A1*, can be observed in unstimulated cultured SFCs is encouraging. However, our sample size is small and a much larger study of patients and controls is required to establish which, if any, gene expression patterns by ME-derived SFCs will have diagnostic utility. Ideally, these studies should be done on selected, uncultured ME cells in order be consistent with a practical diagnostic test.

This study is the first to demonstrate that uNK cells are significantly reduced in ME obtained from endometriosis subjects and that ME-derived SFCs from subjects with endometriosis exhibit significantly impaired decidualization, along with lower expression of *ALDH1A1* than controls. However, there are several limitations that need to be considered. First, the population sizes for our experiments are small, given the prevalence of endometriosis. In order to be confident of our results, we collected multiple ME samples during the first 3 days of the cycle (days 0–2 only) and over multiple cycles for several subjects for the phenotyping studies. We also restricted our functional assays on decidualization capacity using SFCs collected from donors who reported no hormone use, in case hormone use affects decidualization outcomes. In addition, given the small number of samples analyzed by RNA-Seq, we verified our results on *ALDH1A1* using qPCR with SFCs using a larger group of controls and affected subjects. We did not stage our endometriosis subjects based on disease severity, in part because we did not have the confirmed, documented data and because of our restricted sample size. However, all endometriosis diagnoses were confirmed by laparoscopy. It is also important to note that the stage of endometriosis does not necessarily reflect the severity of clinical symptoms a patient presents with (Johnson et al., 2017). It is our goal to develop a diagnostic tool that identifies endometriosis across all stages of endometriosis (particularly, during the early stages); however, having information on the stage of endometriosis would be useful to identify population subsets. Finally, as discussed above, our control group was not laparoscopically confirmed and this may contribute to the small number of subjects that did not ‘classify’ with the result of the controls.

Our studies demonstrate that the ready availability of ME will permit larger studies in the future to develop non-invasive diagnostic methods for endometriosis. These studies will also enable investigation of disease heterogeneity and mechanisms of pathogenesis. Decidualization defects may arise through many different genetic and environmental mechanisms, and these may suggest different approaches to therapy. Indeed it is possible that decidualization defects and gene expression changes in eutopic endometrium may in some cases arise as a result of the presence of endometriosis lesions in the peritoneum, as suggested by studies in the baboon (Fazleabas et al., 2002). In this context it will be of interest to study ME phenotypes before and after surgical or medical treatments and to correlate these findings with the presence or absence of persistent infertility. ME is particularly useful for these types of studies because it can be collected in a non-invasive manner repeatedly over several months. Finally, ME analysis may be very useful in the general evaluation of female infertility, particularly related to decidualization, outside of endometriosis (Gellersen & Brosens, 2014).

## Conclusion

In conclusion, we propose that the routine analysis of ME phenotypes holds promise for understanding and diagnosing endometriosis, as well as potentially other reproductive disorders. This may include the use of proteomic and metabolomic methods, as well as emerging technologies of single cell analysis of freshly isolated cells. Collection using the menstrual cup has proved remarkably useful and should be able to support a variety of such studies, although we believe there is room for development of collection devices that may be tailored to particular analytic questions. We hope that the studies reported here will provoke a wider interest in ME as a clinical and research tool.

## Additional files

Additional file 1: **Table S1.** ME FACS Statistics. **Table S2**. Important Genes Upregulated During 6 h cAMP Stimulation. **Table S3**. Pr Values for Decidualization Time course Experiment. (DOCX 17 kb)

Additional file 2: **Figure S1**. ME-derived SFCs obtained from endometriosis subjects exhibit reduced decidualization capacity when exposed to cAMP+MPA + E2. Time course of IGFBP-1 secretion by vehicle- and 0.5 mM cAMP+ 1 μM MPA+ 10 nM E2-treated ME-derived SFCs isolated from endometriosis (Endo) and control subjects (*n* = 7 control, *n* = 7 endometriosis). Data are shown as IGFBP-1 values for each subject’s SFC culture and the horizontal lines represent group means and vertical lines represent the standard error of the mean. ** posterior probabilities (Pr) < 0.01 *Pr < 0.05. Statistics were performed on log transformed data, as described in the methods. (TIFF 113 kb)

Additional file 3: **Figure S2**. Decidualization capacity can be determined after only 6 h of cAMP stimulation. Log of IGFBP-1 secretion at 6 h, 24 h, and 48 h of cAMP (0.5 mM) stimulation per subject (*n* = 7 control, *n* = 7 endometriosis). (TIFF 170 kb)

## Abbreviations

ALDH1A1: aldehyde dehydrogenase 1 family member A1
cAMP: 8-Bromoadenosine 3′,5′-cyclic monophosphate
E2: 17-beta estradiol
IGFBP-1: insulin growth factor binding protein-1
ME: menstrual effluent
MPA: medroxyprogesterone acetate
MSCs: mesenchymal stem cells
PFA: paraformaldehyde
SFCs: stromal fibroblast cells
