# Supplementary material for: Analysis of menstrual effluent: diagnostic potential for endometriosis
Source: Mol Med. 2018 Mar 19;24:1. doi: 10.1186/s10020-018-0009-6 (PMC6016873; doi:10.1186/s10020-018-0009-6)
Supplement: Supplementary file 1 — Table S1. ME FACS Statistics. Table S2. Important Genes Upregulated During 6 h cAMP Stimulation. Table S3. Pr Values for Decidualization Time course Experiment. (DOCX 17 kb) [file 10020_2018_9_MOESM1_ESM.docx]

| Supplemental Table 1: ME FACS Statistics | | | | | |
| --- | --- | --- | --- | --- | --- |
| Cell Population | Endometriosis | | Controls | | p-value |
|  | Least Squares Mean | Standard Error | Least Squares Mean | Standard Error |  |
| Volume (mL) | 6.08 | 1.14 | 4.75 | 0.93 | 0.3850 |
| WBC | 3.28 | 0.72 | 4.16 | 0.57 | 0.3539 |
| % Cell Population/CD45+ |  |  |  |  |  |
| % CD66b-/CD45+ | 40.65 | 6.46 | 44.50 | 5.04 | 0.6457 |
| % Granulocytes/CD45+ | 56.72 | 6.47 | 55.55 | 5.06 | 0.8892 |
| % Monocytes/CD66b- | 17.87 | 3.40 | 20.55 | 2.66 | 0.5436 |
| % B Cells/CD66b- | 9.40 | 1.44 | 5.68 | 1.11 | 0.0548 |
| % T Cells/CD66b- | 46.10 | 4.30 | 38.33 | 2.89 | 0.1479 |
| % NK Cells/CD66b- | 10.13 | 4.56 | 26.96 | 3.53 | 0.0100 |
| % Cell Population normalized to Live CD45- population |  |  |  |  |  |
| % Live CD45- | 1.87 | 0.54 | 1.56 | 0.30 | 0.6293 |
| % Epithelial Cells/CD45- | 16.11 | 4.85 | 13.70 | 3.12 | 0.6813 |
| % Endothelial Cells/CD45- | 5.67 | 2.43 | 5.53 | 1.62 | 0.9626 |
| % CD326- CD31-/CD45- | 77.84 | 4.76 | 80.78 | 3.21 | 0.6167 |
| % Mesenchymal Stem Cells/CD45- | 28.75 | 8.86 | 49.33 | 4.92 | 0.0535 |

| **Supplemental Table 2:**  **Important Genes Upregulated During 6hr cAMP Stimulation** | | |
| --- | --- | --- |
| Gene | log2FoldChange | P (adjusted) |
| SST | 8.18293962 | 1.03033E-08 |
| IL11 | 4.179836 | 1.16E-12 |
| IGFBP1 | 3.623607 | 1.01E-19 |
| PRL | 3.400631 | 0.00014 |
| FOXO1 | 1.661076 | 2.49E-06 |
| BCL2L11 | 1.340354 | 1.19E-05 |
| WNT5A | 1.210713 | 5.02E-14 |

| **Supplemental Table 3: Pr Values for Decidualization Time course Experiment** | | |
| --- | --- | --- |
|  | | |
|  | | |
| Condition | Time Point | PR Value |
| Endometriosis cAMP vs Control cAMP | 6hrs | 0.0025 |
|  | 24hrs | 0.0045 |
|  | 48hrs | 0.0125 |
| Endometriosis Vehicle vs Control Vehicle | 6hrs | 0.0248 |
|  | 24hrs | 0.0188 |
|  | 48hrs | 0.0308 |
| Endometriosis cAMP+E2+MPA vs Control cAMP+E2+MPA | 6hrs | 0.00225 |
|  | 24hrs | 0.003 |
|  | 48hrs | 0.003 |
| Endometriosis Ethanol Vehicle vs Control Ethanol Vehicle | 6hrs | 0.0025 |
|  | 24hrs | 0.016 |
|  | 48hrs | 0.02 |
