# Supplementary figures and images for: Analysis of menstrual effluent: diagnostic potential for endometriosis
Source: Mol Med. 2018 Mar 19;24:1. doi: 10.1186/s10020-018-0009-6 (PMC6016873; doi:10.1186/s10020-018-0009-6)

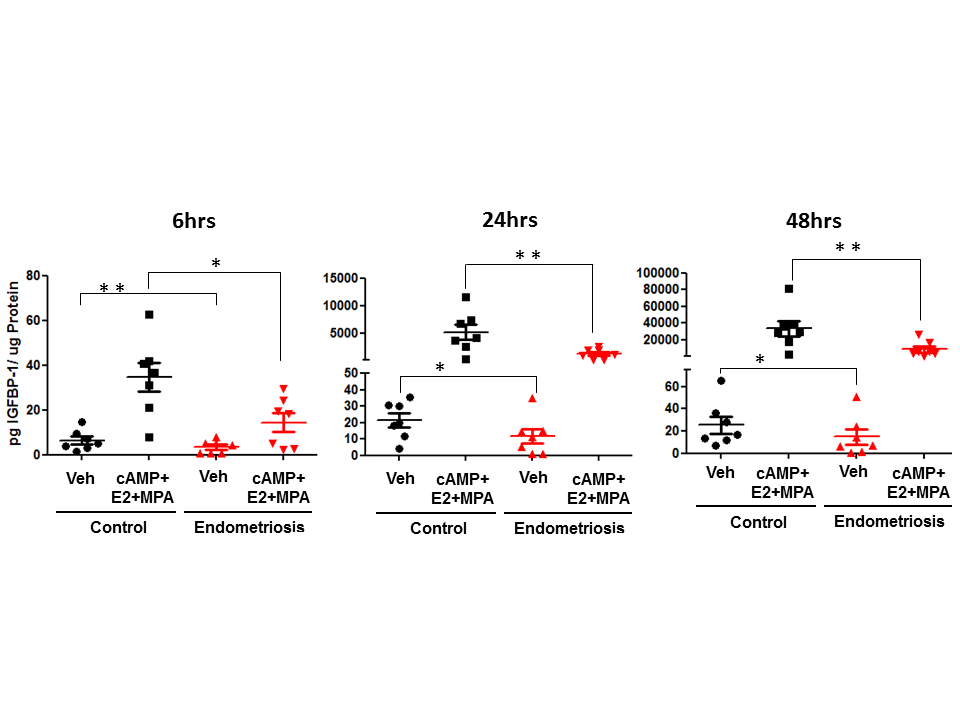

Supplement: Supplementary file 2 — Figure S1. ME-derived SFCs obtained from endometriosis subjects exhibit reduced decidualization capacity when exposed to cAMP+MPA + E2. Time course of IGFBP-1 secretion by vehicle- and 0.5 mM cAMP+ 1 μM MPA+ 10 nM E2-treated ME-derived SFCs isolated from endometriosis (Endo) and control subjects (n = 7 control, n = 7 endometriosis). Data are shown as IGFBP-1 values for each subject’s SFC culture and the horizontal lines represent group means and vertical lines represent the standard error of the mean. ** posterior probabilities (Pr) < 0.01 *Pr < 0.05. Statistics were performed on log transformed data, as described in the methods. (TIFF 113 kb) [file 10020_2018_9_MOESM2_ESM.tif]

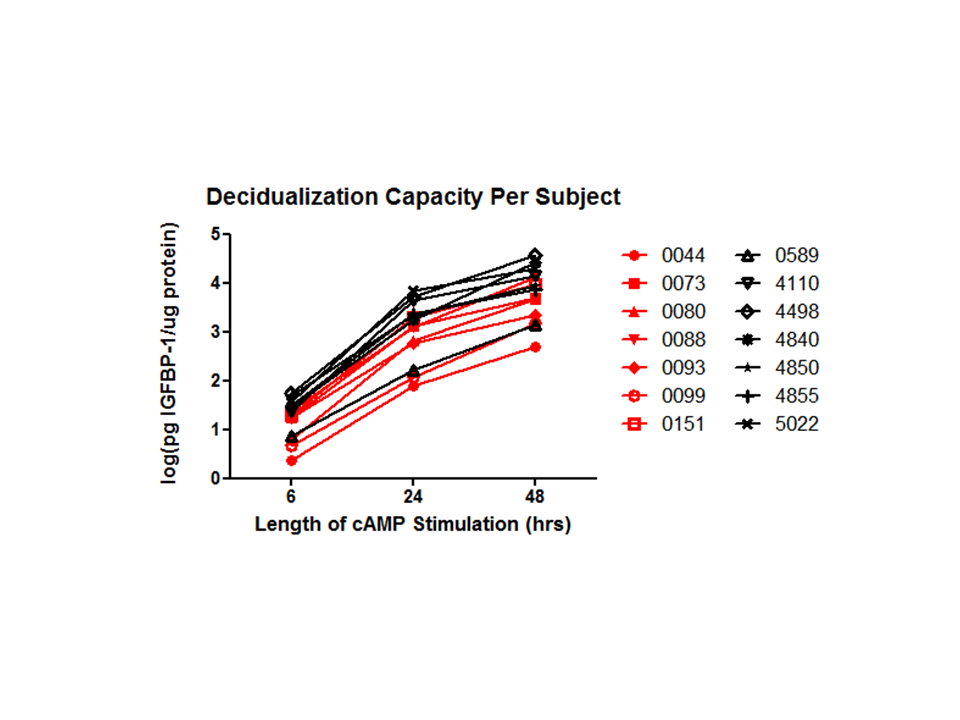

Supplement: Supplementary file 3 — Figure S2. Decidualization capacity can be determined after only 6 h of cAMP stimulation. Log of IGFBP-1 secretion at 6 h, 24 h, and 48 h of cAMP (0.5 mM) stimulation per subject (n = 7 control, n = 7 endometriosis). (TIFF 170 kb) [file 10020_2018_9_MOESM3_ESM.tif]
